# Development of Antibodies with Broad Neutralization Specificities against HIV-1 after Long Term SHIV Infection in Macaques

**DOI:** 10.3390/v12020163

**Published:** 2020-01-31

**Authors:** Nan Gao, Yanxin Gai, Lina Meng, Chu Wang, Xin Zhang, Wei Wang, Chuan Qin, Xianghui Yu, Feng Gao

**Affiliations:** 1 National Engineering Laboratory for AIDS Vaccine, School of Life Sciences, Jilin University, Changchun 130012, China; gaonan15@mails.jlu.edu.cn (N.G.); gaiyx18@mails.jlu.edu.cn (Y.G.); mengln18@mails.jlu.edu.cn (L.M.); wangchu13@mails.jlu.edu.cn (C.W.); xinzhang0913@gmail.com (X.Z.); 2 Institute of Laboratory Animal Science, Chinese Academy of Medical Sciences, Beijing 100021, China; wangw@cnilas.org (W.W.);; 3 Comparative Medicine Center, Peking Union Medical College, Beijing 100021, China; 4 Key Laboratory for Molecular Enzymology and Engineering, the Ministry of Education, School of Life Sciences, Jilin University, Changchun 130012, China; 5 Departments of Medicine, Duke University Medical Center, Durham, NC 27710, USA

**Keywords:** simian/human immunodeficiency virus, neutralizing antibody, specificities, non-human primate, escape mutation

## Abstract

Non-human primates (NHP) are the only animal model suitable to evaluate the protection efficacy of HIV-1 vaccines. It is important to understand how and when neutralizing antibodies (nAbs) with specificities similar to those of human broadly neutralizing antibodies (bnAbs) develop in NHPs. To address these questions, we determined plasma neutralization specificities in two macaques which developed neutralization breadth after long-term simian/human immunodeficiency virus (SHIV) infection and identified neutralization escape mutations by analyzing the *env* sequences from longitudinal plasma samples. Neutralization activities targeting V2, CD4bs, V3 and gp120-gp41 interface only became detectable in week 350 plasma from macaques G1015R and G1020R using 25710 *env* mutants. When mapped with CAP45 *env* mutants, only V2 specificity was detected at week 217 and persisted until week 350 in G1015R. Neutralization escape mutations were found in CD4bs and V2 regions. However, all of them were different from those resistant mutations identified for human bnAbs. These results show that nAbs with specificities similar to human bnAbs are only detectable after long-term SHIV infection and that neutralization escape mutations in macaques are different from those found in HIV-1-infected individuals. These findings can have important implications in the best utilization of the NHP model to evaluate HIV-1 vaccines.

## 1. Introduction

Elicitation of broadly neutralizing antibodies (bnAbs) against globally diverse HIV-1 strains is likely required for a successful vaccine [1,2,3,4]. Broad neutralizing activity can be detected in less than 20% of HIV-1-infected individuals [5,6,7,8]. Importantly, a large number of bnAbs have been isolated and well characterized. Seven conserved sites are targeted by these bnAbs: CD4 binding site (CD4bs), the Env trimer apex in V1V2 region, high-mannose patch in V3, gp120-gp41 interface, fusion peptide (FP), silent face center and membrane-proximal external region (MPER) [3,4,9]. Recently, bnAbs were also explored for their potential applications in HIV-1 prophylactic and therapy [10,11]. Infusion of bnAbs in non-human primates (NHP) and humanized mice prevented acquisition of infection [12,13,14]. However, such potent bnAbs have not been successfully elicited in animal models. A few studies recently showed that potent neutralizing antibodies (nAbs) against autologous viruses with moderate neutralizing breadth could be elicited in different animal models [10,15,16,17,18]. Using the epitope focusing approach, a more recent study showed that the FP-coupled carrier protein immunogens could induce cross-reactive FP-targeted neutralization activities in mice, guinea pigs and NHPs [19,20]. More importantly, mAbs representing the similar neutralization breadth in sera were successfully isolated from several of immunized macaques [21]. These results demonstrate the important roles of bnAbs and possibility to elicit them in NHPs.

Broad neutralizing activity are generally detected after 2–4 years of natural HIV-1 infection in humans [22,23,24]. We recently showed that it took an even longer time (5–6 years) for broad neutralization activities to be detectable in Chinese rhesus macaques infected with simian/human immunodeficiency virus (SHIV) [25]. Importantly, among three different SHIV strains, only SHIV_1157_ induced broad neutralization activities after many years of infection. To determine nAb specificities in sera, the *env* variants containing mutations in the bnAb binding sites that render the viruses resistant have been widely used [26,27,28,29]. This approach allow rapid identification of potential neutralization specificities of nAbs present in tested sera. Using this approach, we previously found that nAbs with V2, CD4bs and V3 specificities, similar to those found in humans, were detectable in SHIV_1157_-infected macaques [25]. However, it was not known when such specificity nAbs developed and if they had selection pressure on the viral population. In this study, we used several additional sets of *env* mutants from other difficult-to-neutralizing tier 2 viruses to determine when different neutralization specificities developed during infection, whether different virus strains affected mapping results, and how CD4bs mutations could impact neutralization susceptibility.

## 2. Materials and Methods 

### 2.1. Ethics Statement

The plasma samples used in this study were archived samples from five long-term SHIV-infected Chinese rhesus macaques reported previously [25,30]. Macaques G1015R and G1020R were intrarectally infected with SHIV_1157ipd3N4_ and followed up for over seven years, with detectable viral loads throughout the infection. Macaques G0802V and G0821R were infected with SHIV_SF162P3_ intravenously and intrarectally, respectively. Macaque G0606R was intrarectally infected with SHIV_CHN19P4_ and the plasma samples from these macaques were collected at 350 weeks post infection. All rhesus macaques were cared for in accordance with the Institutional Animal Care and Use Committee (IACUC) of the Institute of Laboratory Animal Science (approval number: ILAS-VL-2010-004, approval date: October 10, 2008) and the proposals of the Weatherall report [30]. 

### 2.2. Site-Directed Mutagenesis

Mutations in the SHIV_1157ipd3N4_ *env* gene were introduced using the Quick Change Multi Site-Directed mutagenesis kit (Strata gene, La Jolla, CA, USA). Briefly, a pair of oligonucleotide primers containing the desired mutation was used for PCR amplification with KOD DNA polymerase. After amplification, the PCR products were treated with endonuclease Dpn I to digest the parental plasmid DNA template. The treated PCR products were transformed into DH5α competent cell. All *env* mutant clones were confirmed without unintended mutations by sequencing.

### 2.3. Generation of HIV-1 Env Pseudoviruses

The Env pseudoviruses for five *env* genes (1157ipd3N4, X1632, 25710, CAP45 and Ce0217) and their mutants resistant to neutralization by bnAbs were produced by co-transfecting 293T cells with 8 μg of each HIV-1 *env* plasmid and 16 μg of the *env*-deficient HIV-1 backbone clone (pSG3ΔEnv) using Lipofectamine transfection reagent (Invitrogen, Grand Island, NY, USA). Cell culture supernatants were harvested 48 h-72 h following transfection and stored at −80 °C. The 50% tissue culture infectious dose (TCID_50_) for each pseudovirus stock was determined on TZM-bl cells as previously described [25].

### 2.4. Neutralization Assay

Neutralization activities of the plasma samples were determined by the single-round infection of HIV-1 Env-pseudoviruses in TZM-bl cells as described previously [25]. Briefly, plasma samples were first inactivated by incubating at 56 °C for 60 min. After the 1:3 serial diluted plasma samples were incubated with Env-pseudoviruses at 37 °C for 1 h, the mixtures were used to infect TZM-bl cells. Duplicate wells were used for each sample to ensure that results were reproducible. Limited repeats were carried out whenever plasma samples were available. The 50% inhibitory dose (ID_50_) was defined as plasma reciprocal dilution at which relative luminescence units (RLU) were reduced by 50% compared with RLU in virus control wells after subtraction of background RLU in cell control wells. A response was considered positive if the neutralization titer was higher than 1:30.

### 2.5. Single Genome Amplification of the env Gene

Viral RNA was extracted from the plasma samples from G1015R using QiaAmp Viral RNA Mini kit (Qiagen; Valencia, CA, USA), followed by reverse transcription with superscript III reverse transcriptase (Invitrogen, Grand Island, NY, USA). Single genome amplification (SGA) was performed with the newly synthesized cDNA to obtain the complete *env* gene sequences [31]. The *env* genes were amplified using Platinum Taq Polymerase High Fidelity (Invitrogen; Grand Island, NY, USA) with the first round primers Env-F1 (5′-AAGCGGAGACAGCGACGAAGAGC–3′) and Env-R1 (5′-TTGGCCTCACTGATACCCCTACCAAGT-3′) and the second round primers Env-F2 (5′-TAATAGACTAATAGAAAGAGCAGAAGACAGTGGC-3′) and Env-R2 (5′-CCCCTACCAAGTCATCATCTTCCTCATC -3′). The PCR conditions were as follows: one cycle at 94 °C for 2 min; 35 cycles of a denaturing step at 94 °C for 15 s, an annealing step at 64 °C for 30 s, an extension step at 68 °C for 4 min; and one cycle of an additional extension at 68 °C for 10 min. 

### 2.6. Nucleotide Sequence Accession Numbers

The GenBank accession numbers of new SHIV_1157_ *env* sequences reported in this study are MN938237-MN938302.

### 2.7. Statistical Analysis

Statistical analyses were carried out using Graphpad Prism v6 (GraphPad Software, Inc., La Jolla, CA, USA). 

## 3. Results

### 3.1. Development of Neutralization Antibodies with bnAb-Like Specificities after Long Term Infection

Broadly neutralizing activities were detected in two Chinese rhesus macaques (G1015R and G1020R) infected with SHIV_1157_ after 6 years of natural infection in our previous study [25]. Epitope mapping studies with CAP45 and Ce1176 *env* mutants showed that the neutralization breadth was likely contributed by antibodies with V1V2, V3 and CD4bs neutralization specificities by week 321. To understand when these nAbs occurred and if additional neutralization specificities developed during infection, we mapped the neutralization specificities of the longitudinal plasma samples from both macaques. Our previous study showed that 25710 were neutralized by plasma from both G1015R and G1020R from week 27 post infection [25]. Thus, we used a set of eight 25710 *env* mutants that were known for resistant to neutralization by bnAbs targeting V2 [26,27,32], CD4bs [28,33,34], V3 [35] and gp120-gp41 interface [36,37] to determine when bnAb-like neutralization specificities developed in the plasma of G1015R and G1020R. 

**Figure 1:**
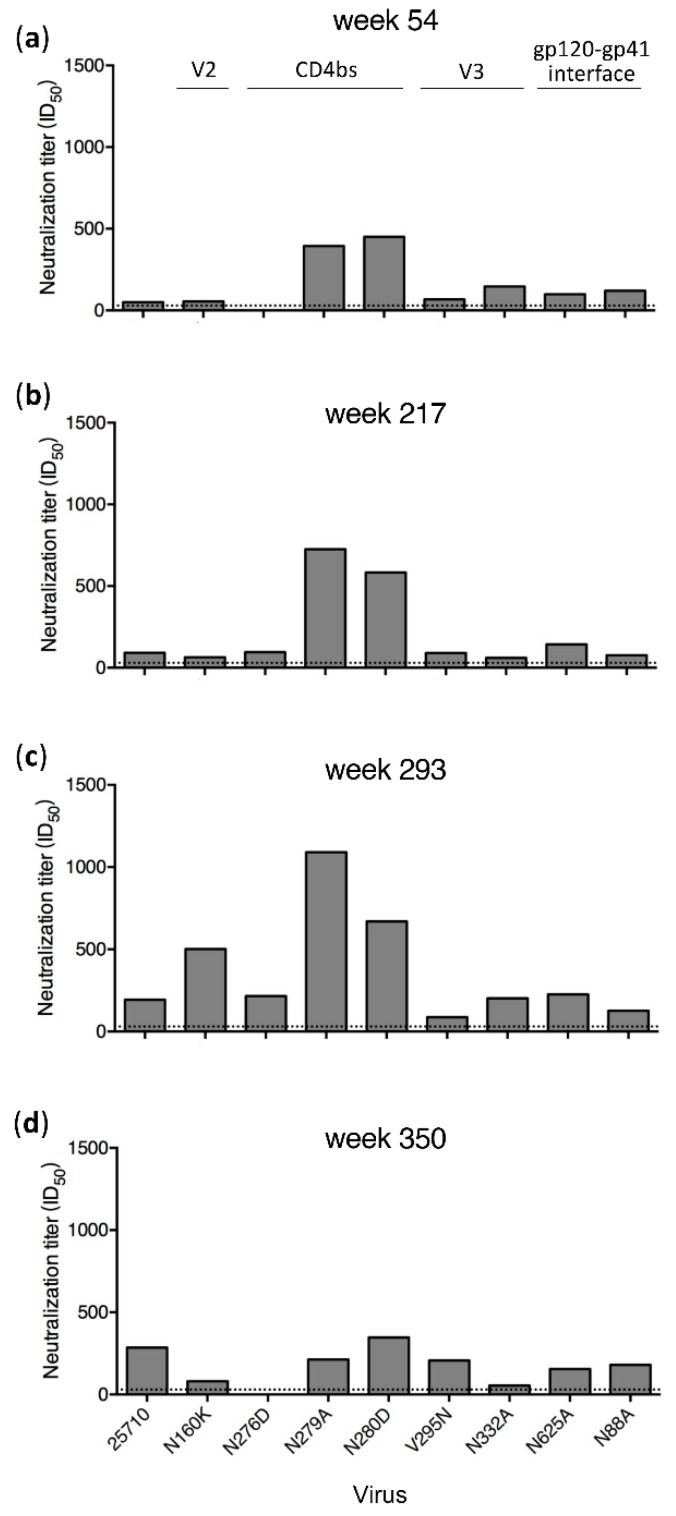
Neutralization specificities of longitudinal plasma from G1020R. Neutralization activities of plasma samples from weeks 54 (**a**), 217 (**b**), 293 (**c**) and 350 (**d**) post infection from G1020R were determined using the wild type 25710 and its *env* mutants (N88A, N160K, N276D, N279A, N280D, V295N, N332A and N625A). The dotted line indicates the lowest dilution (1:30) for the neutralization assay.

**Figure 2:**
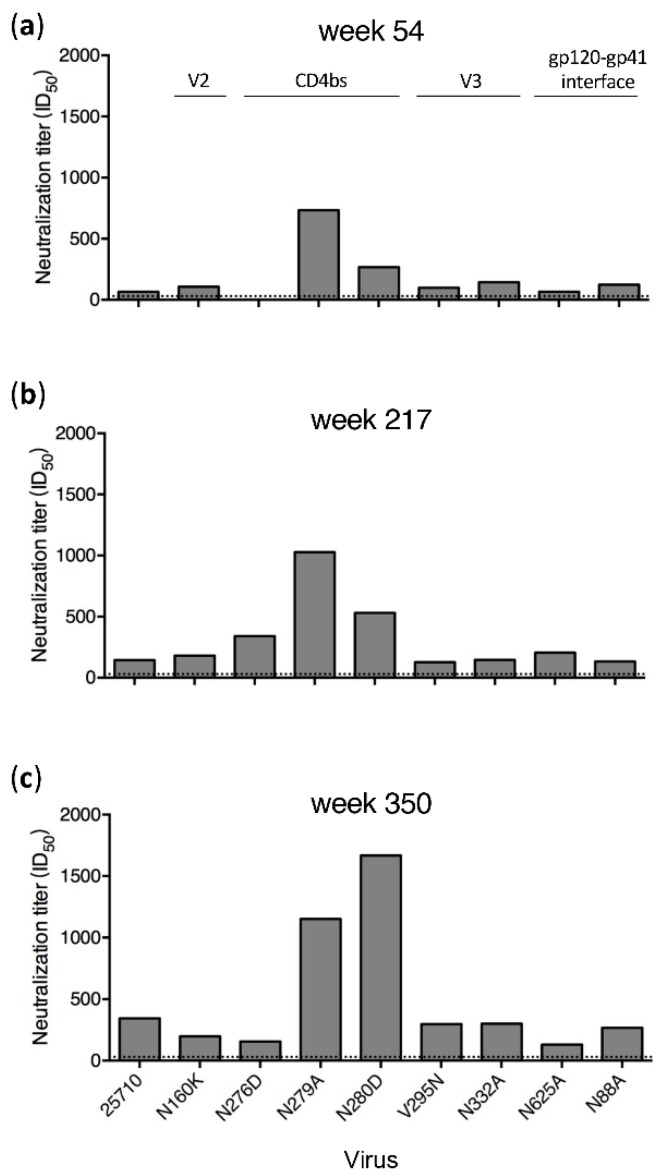
Neutralization specificities of longitudinal plasma from G1015R. Neutralization activities of plasma samples from weeks 54 (**a**), 217 (**b**) and 350 (**c**) post infection from G1015R were determined using the wild type 25710 and its *env* mutants (N88A, N160K, N276D, N279A, N280D, V295N, N332A and N625A). The dotted line indicates the lowest dilution (1:30) for the neutralization assay.

Plasma samples from only four time points (weeks 54, 217, 293 and 350) in G1020R were still available for the mapping analysis (Figure 1). Wild type (wt) 25710 could be neutralized by autologous plasma as early as at week 27 in G1020R [25]. While the neutralization titers against wt 25710 continuously increased from week 54 to week 350 (Figure 1), the neutralization titers of the plasma were reduced by more than half for the N160K (V2), N276D (CD4bs) and N332A (V3) mutants only by week 350 (Figure 1D). While the week 350 plasma could potently neutralize wt 25710 at 1:286 dilution, its neutralization titers were reduced by 91% and 72% for the N332A and N160K mutants, respectively, and it did not neutralize the N276D mutant. The neutralization profile in G1015R was similar to that detected in G1020R (Figure 2). The neutralization against wt 25710 was also detected first at week 27 [25] and its neutralization titers gradually increased over time as in G1020R (Figure 2). Reduced neutralization activities in plasma were not observed for all mutants at week 54 and 217. However, the neutralization titers of the week 350 plasma were reduced by 62% and 55% for the N625A (gp120-gp41 interface) and N276D mutants, respectively. These results suggested that nAbs with V2, CD4bs or V3 specificities were elicited in G1020R while nAbs targeting CD4bs and gp120-gp41 interface were elicited in G1015R. However, nAbs with these neutralization specificities could only be detected at the late infection stage by week 350. 

### 3.2. Disparate Neutralization Profiles among Different Viruses 

**Figure 3:**
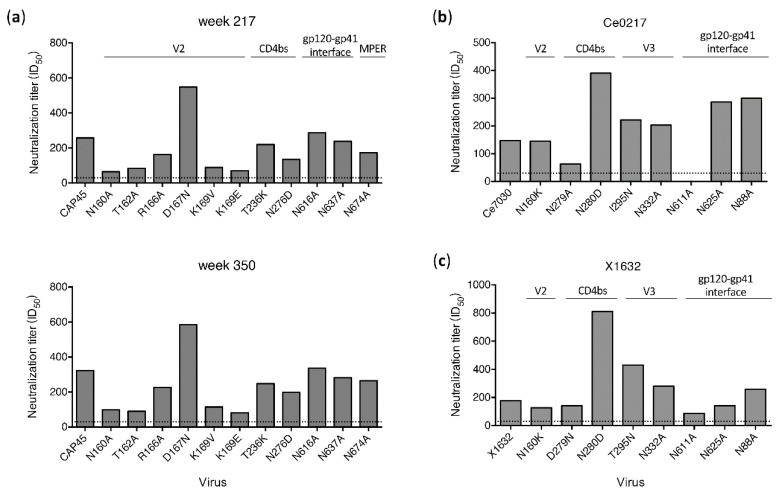
Determination of neutralization specificities of plasma from G1015R using different viruses. (**a**) Neutralization activities of plasma samples from week 217 and 350 post infection were determined using CAP45 and its *env* mutants. Neutralization activities of the week 350 plasma were determined against the Ce0217 (**b**) and X1632 (**c**) mutant sets with mutations in the V2, CD4bs, V3 and gp120-gp41 interface regions. The dotted line indicates the lowest dilution (1:30) for neutralization assay.

When tested with a set of 11 *env* mutants of CAP45, nAbs targeting V2 were identified in the week 321 plasma from G1015R [25]. To determine if different neutralization profiles were present in plasma samples from other time points for which samples were still available for analysis, we mapped potential targeting sites of plasma samples from the earlier (week 217) and later (week 350) time points (Figure 3A). Similar neutralization results were obtained for plasma samples from all three time points. Only weakly reduced neutralization titers (by 65% to 75%) for the N160A, T162A, K169V and K169E mutants were detected. Since all mutations were found in V2, nAbs only with V2 specificities were detected in plasma from all three time points using the CAP45 mutant set. Only two mutation sites (N160K/A and N276D) were shared between CAP45 and 25710, but neutralization sensitivities of both mutants were different between these two viruses. Compared to wt CAP45, the neutralization titers against the N160A mutant were reduced by 75% and 70% by week 217 and 350, respectively (Figure 3A), but the neutralization titer of the N160K mutant of 25710 was only slightly lower compared to wt 25710 (Figure 2). While the N276D mutant was more resistant to neutralization than wt 25710, it was only slightly more resistant than wt CAP45.

To further investigate if nAbs with other specificities could be detected with additional viruses, we test mutant sets from Ce0217 and X1632 (Figure 3B and 3C). Since all but the plasma sample from week 350 in G1015R had been exhausted, only the week 350 plasma was analyzed. When tested with the Ce0217 mutant set, the neutralization titer was reduced by 57% for the CD4bs mutant N279A. It did not neutralize the gp120-gp41 interface mutant N611A (Figure 3B). When tested with the X1632 mutant set, the plasma only showed weakly reduced neutralization activity (by 51%) against the N611A mutant. These results showed that the gp120-gp41 interface was most likely targeted by nAbs in the week 350 plasma from G1015R. 

Taken together, these results indicate that when different viruses are used to map potential neutralization specificities, results can vary from one virus to another. Only a subset of potential epitopes can be identified when one virus mutant set is used to determine nAb specificities. Thus, overall neutralization specificities will be better reflected by using multiple different *env* mutant sets. 

### 3.3. Neutralization Resistant Mutations Were Selected by Autologous Antibodies

All potential epitopes above were identified using heterologous tier 2 viruses. To determine if these mutation sites were indeed targeted by autologous nAbs in G1015R, we examined the *env* gene sequences from three time points (weeks 27, 214 and 350), for which there were still enough plasma samples for single genome amplification (SGA) analysis. No samples from G1020R were available for similar analysis. The 10 sequences from the stock were homogenous, with only rare random mutations (Figure S1). Since viruses with CD4bs mutations (N276D and N279A), V2 mutations (N160A, T162A, K169V and K169E) and gp120-gp41 interface mutations (N611A and N625A) were found resistant to neutralization by week 350 plasma in G1015R, these regions were carefully examined. 

**Figure 4:**
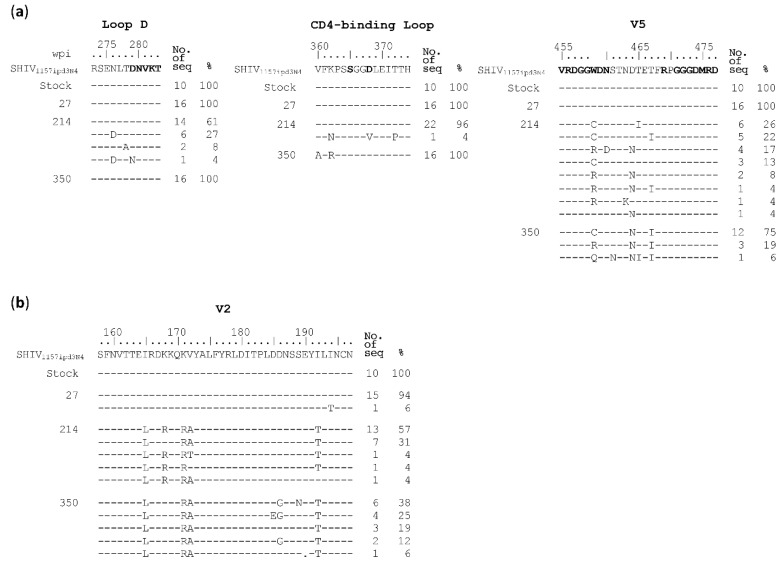
Identification of neutralization escape mutations in the *env* gene from longitudinal samples in G1015R. Amino acid sequences of (**a**) CD4 binding site (Loop D, CD4-binding loop and V5) and (**b**) V2 region from the viral stock and weeks 27, 214 and 350 post infection were compared to the SHIV_1157_ reference sequence. The amino acids associated with CD4 binding are shown in bold letters. The amino acid positions are based on the HIV-1 HXB2 sequence.

Viral sequences at week 27 were as homogenous as those in the viral stock, except that two mutations were found in the heptad repeat regions in gp41. Only one mutation in the N-terminal heptad repeat was subsequently fixed in the later time points (Figure S1). The N276D mutation in loop D was detected in 7 out of 23 sequences at week 214 but became undetectable at week 350 (Figure 4A). No mutations at position 280 were detected in any sequences while one reversion mutation D279N was found in only 1 out of 23 sequences at week 214. Since CD4bs bnAbs also bind to the CD4 binding-loop and the V5 region [33,38,39,40], we next examined if mutations could be detected in those two regions. In the CD4-binding loop, only one sequence with three amino acid substitutions were found among 23 sequence at week 214, while all 16 sequences had two amino acids substitution at week 350 (Figure 4A). However, none of these substitutions were at major contact sites for CD4bs bnAbs [35,38,39,40]. There were two amino acids (WD) insertion after position 459 in the SHIV_1157_ Env, compared to the HXB2 sequence. At the position W459′, all but one sequences were substituted by R (W459′R; 35%) or C (W459′C; 61%) at week 214. The N460D mutation were found in 4 of 23 (17%) sequences of the week 214 sequences. By week 350, a new W459′Q mutation was found in 1 of 16 sequences. Four amino acid substitutions in V2 region were detected among the majority sequences in the week 214 sample and fixed in all sequences in the week 350 sample (Figure 4B). However, none of them were similar to those mutations (N160A, T162A, K169V/E) that were found to be resistant to known V2 bnAbs. No mutations at position 332 and known sites associated with resistance to gp120-gp41 interface bnAbs were found in all sequences from three time points (Figure S1). 

**Figure 5:**
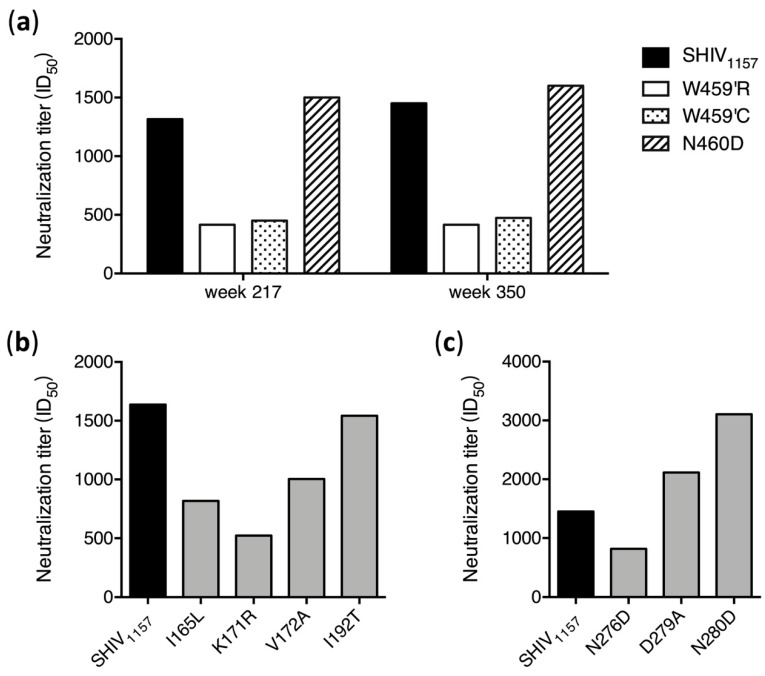
Neutralization susceptibility of escape mutants to autologous plasma. (**a**) Neutralization sensitivity of the *env* mutants with mutations in V5 was determined using the week 217 and week 350 plasma samples from G1015R. Neutralization sensitivities of the *env* mutants with mutations in V2 (**b**) and loop D (**c**) were determined using the week 350 plasma from G1015R.

To investigate if these predominant mutations were selected by nAbs, we introduced them into the SHIV_1157_ *env* gene. Both W459′R and W459′C mutants were much more resistant to week 217 plasma than the wt SHIV_1157_, while the N460D mutant was similarly sensitive to neutralization as wt SHIV_1157_ (Figure 5A), which could explain the reason this mutation disappeared from those sequences of week 350. This demonstrated that both W459′R and W459′C mutations were selected by nAbs targeting V5 in G1015R. The similar results were observed with week 350 plasma (Figure 5A). When four V2 mutants were tested, the K171R mutant was much more resistant to neutralization than wt SHIV_1157_, but both I165L and V172A mutants were only slightly more resistant (Figure 5C). The I192T mutant was similarly neutralized as wt SHIV_1157_. This suggests that the three mutations close to each other (at positions 165–172) in V2 were under the selection pressure by nAbs targeting V2. 

### 3.4. N279A and N280D Mutations Render Env Mutants More Sensitive to Neutralization

**Figure 6:**
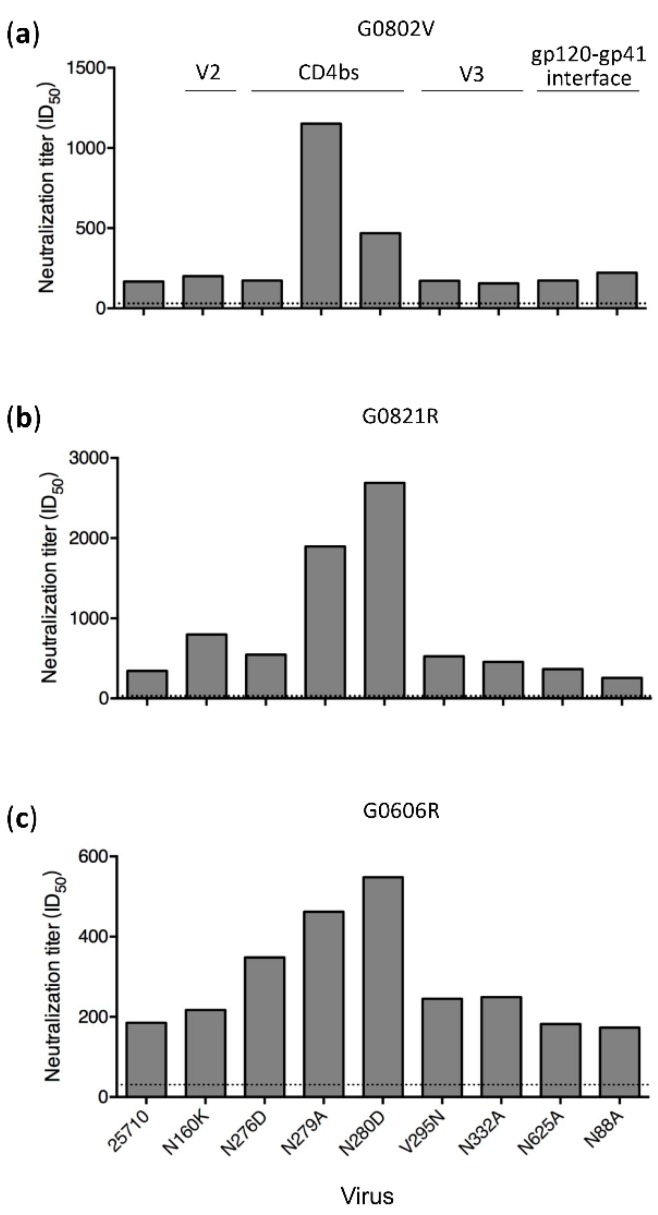
High levels of neutralization sensitivity of loop D mutants to plasma from macaques infected with other SHIV strains. Neutralization activities were determined for the plasma samples from macaques G0802V (**a**) and G0821R (**b**) which were infected with SHIV_SF162P3_, and macaque G0606R (**c**) which was infected with SHIV_CHN19P4_. The neutralization assay was performed using the 25710 *env* mutant set. The dotted line indicates the lowest dilution (1:30) for the neutralization assay.

Among all the mutants tested, the N279A and N280D mutations in 25710 rendered the viruses more sensitive to neutralization by plasma from all time points in both G1015R and G1020R macaques (Figure 1 and Figure 2). While the neutralization titers against wt 25710 in both macaques continuously increased over time, the neutralization titers against both N279A and N280D mutants remained at the similar levels. The N280D mutation also rendered the Ce0217 and X1632 viruses more sensitive to the week 350 plasma in G1015R, while the N279A mutant of Ce0217 was more resistant (Figure 3B,C). The reversion mutation D279N in X1632 had no effect on the neutralization susceptibility (Figure 3C). Both N279A and N280D mutations in 25710 also made the viruses more sensitive to neutralization in macaques infected with SHIV_SF162P3_ or SHIV_CHN19P4_ (Figure 6), in which only low titer neutralization activities with limited breadth were detected after long-term infection [25]. 

**Figure 7:**
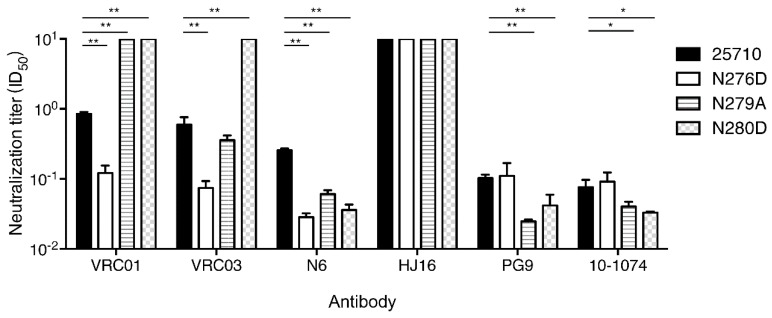
Neutralization susceptibility of loop D mutants to various bnAbs. The neutralization sensitivity of loop D mutants (N276D, N279A and N280D) of 25710 was determined using bnAbs that target CD4bs (VRC01, VRC03, N6 and HJ16), V2 apex (PG9) and V3 glycan (10–1074). Statistical analysis was performed by unpaired t test (*: 0.05 > *p* > 0.01; **: *p* < 0.01).

To investigate if both mutations made viruses universally sensitive to neutralization, we tested them against six bnAbs targeting different epitopes. Among three loop D mutants tested, the N276D mutant were more sensitive to three CD4bs bnAbs (VRC01, VRC03 and N6). However, the N279A was more resistant to VRC01 than wt 25710, similarly sensitive to VRC03, but more sensitive to N6 (Figure 7). The N280D mutant were more resistant to both VRC01 and VRC03 but more sensitive to N6. The wt 25710 and three mutants were not neutralized by another CD4bs bnAb HJ16, which had a different binding pattern compared to other CD4bs bnAbs [33,41]. When V1V2 bnAb PG9 and V3 bnAb 10–1074 were tested, the N276D mutant was similarly neutralized as wt 25710, but both N279A and N280D mutants were more sensitive to neutralization. These results showed that the N279A and N280D mutations had different impacts on resistance to neutralization by various CD4bs bnAbs, but were more sensitive to neutralization by bnAbs targeting V1V2 or V3. The N276D, N279A and N280D mutations in loop D generally have low binding affinity and are more resistant to CD4bs bnAbs, but the differences in binding and neutralizing capabilities vary significantly [34,40,42,43]. We also found that some mutations in loop D were resistant to CH235 lineage bnAbs but more sensitive to CH103 lineage bnAbs than the wild type virus, although both CH103 and CH235 lineage bnAbs targeted the same CD4 binding site [28,44]. These results suggest that the same mutations in loop D can have disparate impacts on neutralization susceptibility to different CD4bs bnAbs. Thus, it is not surprising to see different neutralization sensitivities of some loop D mutants to different CD4bs bnAbs, especially for N6 and HJ16 which had binding patterns different from those of VRC01-like bnAbs [33,41]. 

We next introduced them individually into the SHIV_1157_ genome to study how these CD4 binding-loop mutations in SHIV_1157_ affected neutralization susceptibility to autologous plasma. The N276D mutant was slightly more resistant to week 350 plasma than wt SHIV_1157_ (Figure 5C). This was consistent with the mapping results with the 25710 mutants (Figure 2), suggesting that the transient presence of the N276D mutation at week 214 was indeed selected by nAbs targeting loop D in G1015R (Figure 4A). However, both D279A and N280D mutants were more sensitive to week 350 plasma from G1015R. This suggests that the D279A or N280D mutation would be more sensitively neutralized by the autologous plasma than the wt SHIV_1157_ in G1015R, although either D279A or N280D mutation was not detected in G1015R.

## 4. Discussion

We previously found that broad neutralization activities were detected in Chinese rhesus macaques only after six years of infection [25]. Now we determined the neutralization specificities using different sets of Env mutants in bnAb epitopes in two macaques which developed broad neutralization activities. We found that nAbs with specificities similar to human bnAbs were only detectable after 4–5 years of infection. This indicates that the long mature time of broad neutralization is also required in Chinese rhesus macaques as in humans [23,24]. 

Resistance of HIV-1 mutants with known bnAb escape mutations to neutralization by plasma was only detected after many years of infection in both macaques (G1015R and G1020R). HIV-1 25710 was similarly neutralized by plasma from G1015R and G1020R. However, mutations in bnAb epitopes in 25710 only render the viruses more resistant to neutralization by both macaque plasma after seven years of infection. This is in good agreement with our previous observation that broad neutralization activities were detected after 5–6 years of infection in both macaques [25]. This suggests that the bnAb specificities develop only after long maturation process in both macaques. Our results also showed that when the nAbs with bnAb-like specificities developed, they could persist over 130 weeks (Figure 3A). However, the limited mutations in each virus may not detect all bnAb-like specificities in any given macaques. More inclusive analysis of mutants is needed to better understand the majority of bnAb-like specificities in each animal. The long maturation of broad neutralization in NHPs will have important implications in use of the NHP model. This suggests that an immunization strategy over a long period of time may be required to elicit bnAbs that are most likely required for development of a successful HIV-1 vaccine. 

Escape mutations were identified in V2, loop D and V5 regions by analyzing of SGA sequences from longitudinal plasma samples in G1015R. When they were introduced into wt SHIV_1157_ *env* gene, the mutants were more resistant to neutralization by autologous plasma. Similar V5-dependent neutralizing activity was reported in SHIV_SF162P3N_-infected rhesus macaques [45,46]. Because the samples from G1015R between week 27 and week 214 were not available for analysis, we could not precisely determine when these mutations were selected between those two time points. The results suggest that those regions often targeted by bnAbs in humans are also targeted in macaques. However, the most of the mutations in these regions are different between viruses in humans and macaques. This suggests that the mechanisms for viruses to escape broad neutralizing antibodies in macaques are different from those in humans, although the similar sites are targeted in both humans and macaques.

The neutralization specificities varied significantly when mutation sets from different *env* genes were analyzed. This suggests that different *env* backbones can have significant impacts on neutralization susceptibility even when the same mutations are present. Thus, when a neutralization specificity is detected with one virus, it is likely that such neutralization specificity is elicited. However, when a neutralization specificity is not detected with one virus, it may be detected with other viruses. Therefore, multiple heterologous viruses are required to better determine potential neutralization specificities in a sample. 

One other interesting observation is that the N279A and N280D mutations rendered viruses more sensitive to neutralization by plasma. This was found in different SHIV strains and in different macaques irrespective of presence or absence of broad neutralization activities. When tested against bnAbs targeting different epitopes, we found that both mutations had various impacts on neutralization susceptibility to different CD4bs specific bnAbs, but rendered them more sensitive to neutralization by bnAbs with the V1V2 or V3 specificity. Interestingly, none of these mutations were detected in the macaque. This suggests that if either mutation is selected by nAbs in the macaque, it could be rapidly neutralized by nAbs, or its high sensitivity to neutralization may enhance the maturation process of certain bnAb lineages, possibly due to the high affinity to CD4bs bnAbs, as the cooperation between CH235 and CH103 CD4bs bnAb lineages to drive further maturation of CH103 lineage Abs in our previous report [28]. This is also in agreement with the results from a study in which the N279D mutation could increase Ab affinity maturation by decreasing the dependence of Abs on this amino acid and likely played a role in the development of neutralization breadth to the CD4 binding sites [27].

The findings of long maturation time of broad neutralization activities, different escape mutations from those selected by human bnAbs and high levels of neutralization activities against some CD4bs mutations in SHIV-infected Chinese rhesus macaques will have important implications in the best unitization of NHP model to evaluate HIV vaccines. Isolation and characterization of monoclonal antibodies with broad neutralization activities from these animals can further ratify these new findings. 

## Supplementary Materials

The following are available online at https://www.mdpi.com/1999-4915/12/2/163/s1, Figure S1 Alignment of amino acid sequences of the env genes from longitudinal samples in SHIV_1157_ infected macaque, related to Figure 4. Amino acid sequences of the complete *env* gene from the viral stock and weeks 27, 214 and 350 post infection are compared to the SHIV_1157_ reference sequence. Amino acid substitutions are denoted as upper-case letters. Dashes represent identical amino acid residues and dots represent the deletions. Hypervariable regions (V1 through V5), loop D, CD4-binding loop and gp120-gp41 interface are indicated by gray shade areas. Residues which are known to interact with CD4 molecular are underlined.
