# Supplementary material for: Development of Antibodies with Broad Neutralization Specificities against HIV-1 after Long Term SHIV Infection in Macaques
Source: Viruses. 2020 Jan 31;12(2):163. doi: 10.3390/v12020163 (PMC7077270; doi:10.3390/v12020163)
Supplement: Supplementary file 1 [file viruses-12-00163-s001.pdf]

```
                                                                                                                                                                     V1                              V2
SHIV1157.S        YQHLWRWGWRWGIMLLGMLMICSASEKLWVTVYYGVPVWKEAKTTLFCASNAKAYEKEVHNIWATHACVPTDPNPQEIVLGNVTENFNMWKNDMVDQMHEDIISLWDQSLKPCVKLTSLCVTLKCSNFTGKSNVTYKGDMEVKNCSFNVTTEIRDKKQKVYALFYRLDITPLDDNSSEYILINCNSSTITQACPKVNFDPI  201
SHIV1157.S.01     ---------------------------------------------------------------------------------------------------------------------------------------------------------------------------------------------------------
SHIV1157.S.02     ---------------------------------------------------------------------------------------------------------------------------------------------------------------------------------------------------------
SHIV1157.S.03     ---------------------------------------------------------------------------------------------------------------------------------------------------------------------------------------------------------
SHIV1157.S.04     ---------------------------------------------------------------------------------------------------------------------------------------------------------------------------------------------------------
SHIV1157.S.05     ---------------------------------------------------------------------------------------------------------------------------------------------------------------------------------------------------------
SHIV1157.S.06     ---------------------------------------------------------------------------------------------------------------------------------------------------------------------------------------------------------
SHIV1157.S.07     ---------------------------V-------------------------------------------------------------------------------------------------------------------------------------------------------------------------
SHIV1157.S.08     ---------------------------------------------------------------------------------------------------------------------------------------------------------------------------------------------------------
SHIV1157.S.09     ---------------------------------------------------------------------------------------------------------------------------------------------------------------------------------------------------------
SHIV1157.S.10     ---------------------------------------------------------------------------------------------------------------------------------------------------------------------------------------------------------

SHIV1157.w27.01   ---------------------------------------------------------------------------------------------------------------------------------------------------------------------------------------------------------
SHIV1157.w27.02   ---------------------------------------------------------------------------------------------------------------------------------------------------------------------------------------------------------
SHIV1157.w27.03   --------------------------------------------------------------------------------R---------------------V--------------------------------------------------------------------------------------------------
SHIV1157.w27.04   -------------------------------------------------------------------------------------------------------------------------------------------------------------------------------------T-------------------
SHIV1157.w27.05   ---------------------------------------------------------------------------------------------------------------------------------------------------------------------------------------------------------
SHIV1157.w27.06   ---------------------------------------------------------------------------------------------------------------------------------------------------------------------------------------------------------
SHIV1157.w27.07   ---------------------------------------------------------------------------------------------------------------------------------------------------------------------------------------------------------
SHIV1157.w27.08   ---------------------------------------------------------------------------------------------------------------------------------------------------------------------------------------------------------
SHIV1157.w27.09   ---------------------------------------------------------------------------------------------------------------------------------------------------------------------------------------------------------
SHIV1157.w27.10   ---------------------------------------------------------------------------------------------------------------------------------------------------------------------------------------------------------
SHIV1157.w27.11   ---------------------------------------------------------------------------------------------------------------------------------------------------------------------------------------------------------
SHIV1157.w27.12   ----------------------------------------------------------------------------------------------------------------------------------------------------------------------------------------------R----------
SHIV1157.w27.13   ---------------------------------------------------------------------------------------------------------------------------------------------------------------------------------------------------------
SHIV1157.w27.14   ---------------------------------------------------------------------------------------------------------------------------------------------------------------------------------------------------------
SHIV1157.w27.15   ----------------------------------------------------------------------------------------------------------------------------------------------------------------------------------------------R----------
SHIV1157.w27.16   ---------------------------------------------------------------------------------------------------------------------------------------------------------------------------------------------------------

SHIV1157.w214.01  --------------------------------------------------D-----------------------------------------N----------------------------------V-E-G-F--N-AT-----------L--R--RA-------------------T--------L------------
SHIV1157.w214.02  --------------------------------------------------D-----------------------------------------N----------------------------------V-E-G-F--N-AT-----------L--R--RA-------------------T--------L------------
SHIV1157.w214.03  --------------------------------------------------D-----------------------------------------N----------------------------------V-E-G-F--N-AT-----------L--R--RA-------------------T--------L------------
SHIV1157.w214.04  --------------------------------------------------D-----------------------------------------N----------------------------------V-E-G-F--N-AT-----------L--R--RA-------------------T--------L------------
SHIV1157.w214.05  -------------------------G------------------------D-----------------------------------------N----------------------------------V-E-G-F--N-AT-----------L--R--RT-------------------T--------L------------
SHIV1157.w214.06  --------------------------------------------------D-----------------------------------------N----------------------------------V-E-G-F--N-AT-----------L--R--RA----------------------------L------------
SHIV1157.w214.07  --------------------------------------------------D-----------------------------------------N----------------------------------V-K-G-F--N-AT-----------L-----RA-------------------T--------L------------
SHIV1157.w214.08  --------------------------------------------------D-----------------------------------------N----------------------------------V-E-G-F--N-AT-----------L-----RA-------------------T--------L------------
SHIV1157.w214.09  ------------------V-------------------------------D--------------------------------------G--N----------------------------------V-E-G-F--N-AT-----------L-----RA-------------------T--------L------------
SHIV1157.w214.10  --------------------------------------------------D-----------------------------------------N----------------------------------V-E-G-F-HN-AT-----------L-----RA-------------------T--------L------------
SHIV1157.w214.11  --------------------------------------------------D-----------------------------------------N----------------------------------V-E-GKF--N-AT-----------L--R--RA-------------------T--------L------------
SHIV1157.w214.12  --------------------------------------------------D-----------------------------------------N----------------------------------V-E-G-F--N-AT-----------L-----RA-------------------T--------L------------
SHIV1157.w214.13  -------------------------G------------------------D-----------------------------------------N----------------------------------V-E-G-F-HN-AT-----------L--R--RA-------------------T--------L------------
SHIV1157.w214.14  --------------------------------------------------D-----------------------------------------N----------------------------------V-E-G-F--N-AT-----------L--R--RA-------------------T--------L------------
SHIV1157.w214.15  --------------------------------------------------D-----------------------------------------N----------------------------------V-E-G-F--N-AT-----------L--R--R--------------------T--------L------------
SHIV1157.w214.16  --------------------------------------------------D-----------------------------------------N------------------------------R---V-E-G-F--N-AT-----------L--R--RA-------------------T--------L------------
SHIV1157.w214.17  --------------------------------------------------D-----------------------------------------N----------------------------------V-E-G-F--N-AT-----------L--R--RA-------------------T--------L------------
SHIV1157.w214.18  --------------------------------------------------D-----------------------------------------N----------------------------------V-E-G-F--N-AT-----------L--R--RA-------------------T--------L------------
SHIV1157.w214.19  -------------------------Q------------------------D-----------------------------------------N------------------------------R---V-E-G-F--N-AT-----------L--R--RA-------------------T--------L------------
SHIV1157.w214.20  --------------------------------------------------D-----------------------------------------N----------------------------------V-E-G-F--N-AT-----------L--R--RA-------------------T--------L------------
SHIV1157.w214.21  --------------------------------------------------D-----------------------------------------N----------------------------------V-E---F--N-AT-----------L-----RA-------------------T--------L------------
SHIV1157.w214.22  --------------------------N---------------..........----------------------------------------N----------------------------------V-E-D-F--N-AT-----------L--R--RA-------------------T--------L------------
SHIV1157.w214.23  ------G-------------------------------------------D-----------------------------------------N----------------------------------V-E---F--N-AT-----------L-----RA-------------------T--------L------------

SHIV1157.w350.01  --------------------------N-------------D---------D-----------------------------------------N------------------------------R-KDLNTT....-QKAT-----------L-----RA------------EG-----T--------L------------
SHIV1157.w350.02  -------------------L------N-------------D---------D-----------------------------------------N--------------------------------R-A-......-QEAT-----------L-----RA-------------------T--------L------------
SHIV1157.w350.03  -------------------L------N-----------------------D-----------------------------------------N------------------------------R-KDLNTTY..QRDNFT-----------L-----RA-------------G--N--T--------L------------
SHIV1157.w350.04  -------------------L------N-----------------------D-----------------------------------------N--------------------------------R-A-......-QEAT-----------L-----RA-------------G-----T--------L------------
SHIV1157.w350.05  --------------------------N-----------------------D-----------------------------------------N------------------------------R-KDLNTTY..QRDNFT-----------L-----RA-------------G--N--T--------L------------
SHIV1157.w350.06  --------------------------N-----------------------D-----------------------------------------N------------------------------R-KDLNTTY..QRDNFT-----------L-----RA-------------G--N--T--------L------------
SHIV1157.w350.07  -----------------I--------N-----------------------D-----------------------------E-----------N------------------------------N--DV-R-N-SIHD-AT-----------L-----RA-------------G-----T--------L------------
SHIV1157.w350.08  --------------------------N-----------------------D-----------------------------------------N------------------------------R-KDLNTTY..QRDNFT-----------L-----RA-------------G--N--T--------L------------
SHIV1157.w350.09  --------------------------N-----------------------D-----------------------------------------N------------------------------R-KDLNTTY..QRDNFT-----------L-----RA-------------G--N--T--------L------------
SHIV1157.w350.10  -H------------------------N-----------------------D-----------------------------------------N------------------------------R-KDLNTTY..QRDNFT-----------L-----RA-------------G--N--T--------L------------
SHIV1157.w350.11  --------------------------N-------------D---------D-----------------------------------------N------------------------------R-KDLNTT....-QKAT-----------L-----RA------------EG-----T--------L------------
SHIV1157.w350.12  -------------------L------N-----------------------D---------------------------------------E-N--------------------------------R-A-......-QEAT-----------L-----RA-------------------T--------L------------
SHIV1157.w350.13  -------------------L------N-------------D---------D-----------------------------------------N------------------------------N--DV-R-N-SIHD-AT-----------L-----RA-----------------.-T--------L------------
SHIV1157.w350.14  -------------------L------N-----------------------D--------------------------------------------------------------------------R-V--R.....DNFT-----------L-----RA-------------------T--------L------------
SHIV1157.w350.15  -------------------L------N-----------------------D-------------------------------I---------N--------------------------------R-A-......-QEAT-----------L-----RA------------EG-----T--------L------------
SHIV1157.w350.16  --------------------------N-------------D---------D-----------------------------------------N------------------------------E---V--......DNFT-----------L-----RA------------EG-----T--------L------------
```

```
                                                                               Loop D                        V3                                            CD4-binding loop                      V4
SHIV1157.S       PIHYCAPAGYAILKCNNKTFNGTGPCHNVSTVQCTHGIKPVVSTQLLLNGSLAEGEIIIRSENLTDNVKTIIVHFNESVEITCTRPNNNTRKSISIGPGQAIYATGDIIGDIRQAHCNISKENWNKTLQWVRGKLKEHFPNKTIVFKPSSGGDLEITTHSFNCRGEFFYCNTSKLFNSTDNSTHMGTENNTIITIPCRIKQ  402
SHIV1157.S.01    ---------------------------------------------------------------------------------------------------------------------------------------------------------------------------------------------------------
SHIV1157.S.02    ---------------------------------------------------------------------------------------------------------------------------------------------------------------------------------------------------------
SHIV1157.S.03    ----------------------------------------------------V--------------------------------------------------------------------------------------------------------------------------------------------------------
SHIV1157.S.04    ---------------------------------------------------------------------------------------------------------------------------------------------------------------------------------------------------------
SHIV1157.S.05    ---------------------------------------------------------------------------------------------------------------------------------------------------------------------------------------------------------
SHIV1157.S.06    ---------------------------------------------------------------------------------------------------------------------------------------------------------------------------------------------------------
SHIV1157.S.07    ---------------------------------------------------------------------------------------------------------------------------------------------------------------------------------------------------------
SHIV1157.S.08    ---------------------------------------------------------------------------------------------------------------------------------------------------------------------------------------------------------
SHIV1157.S.09    ---------------------------------------------------------------------------------------------------------------------------------------------------------------------------------------------------------
SHIV1157.S.10    ---------------------------------------------------------------------------------------------------------------------------------------------------------------------------------------------------------

SHIV1157.w27.01  ---------------------------------------------------------------------------------------------------------------------------------------------------------------------------------------------------------
SHIV1157.w27.02  ---------------------------------------------------------------------------------------------------------------------------------------------------------------------------------------------------------
SHIV1157.w27.03  ---------------------------------------------------------------------------------------------------------------------------------------------------------------------------------------------------------
SHIV1157.w27.04  ---------------------------------------------------------------------------------------------------------------------------------------------------------------------------------------------------------
SHIV1157.w27.05  ---------------------------------------------------------------------------------------------------------------------------------------------------------------------------------------------------------
SHIV1157.w27.06  ---------------------------------------------------------------------------------------------------------------------------------------------------------------------------------------------------------
SHIV1157.w27.07  ----------------------------------------------------------------------------------------------------------------------------S----------------------------------------------------------------------------
SHIV1157.w27.08  ---------------------------------------------------------------------------------------------------------------------------------------------------------------------------------------------------------
SHIV1157.w27.09  ---------------------------------------------------------------------------------------------------------------------------------------------------------------------------------------------------------
SHIV1157.w27.10  ---------------------------------------------------------------------------------------------------------------------------------------------------------------------------------------------------------
SHIV1157.w27.11  ---------------------------------------------------------------------------------------------------------------------------------------------------------------------------------------------------------
SHIV1157.w27.12  ---------------------------------------------------------------------------------------------------------------------------------------------------------------------------------------------------------
SHIV1157.w27.13  ---------------------------------------------------------------------------------------------------------------------------------------------------------------------------------------------------------
SHIV1157.w27.14  -----------------------------------------------------------------------------------------------------------------------------------------------------------------------------------G---------------------
SHIV1157.w27.15  ----------------------------------------------------------------------------------------------N----------------------------------------------------------------------------------------------------------
SHIV1157.w27.16  ------------------------------------------------------------------------------------------------------------------------------------E--------------------------------K-----------------------------------

SHIV1157.w214.01 ------------------------------------------------------E-------------------L-------------------R------L---------------------------R--------------------------------------------------------D---D--.--------
SHIV1157.w214.02 ------------------------------------------------------E-------D-----------L-------------------R------L-------------------G-------R--------------------------------------------------------D---D-----------
SHIV1157.w214.03 ------------------------------------------------------E-------D-----------L-------------------R------L-------------------G-------R-----------------------------------------------N-G-----D---D-----------
SHIV1157.w214.04 ------------------------------------------------------E-------D-----------L-------------------R------L-------------------G-------R--------------------------------------------------------D---D-----------
SHIV1157.w214.05 ------------------------------------------------------E-------D-----------L-------------------R------L-------------------G-------R--------------------------------------------------------D---D-----------
SHIV1157.w214.06 ------------------------------------------------------E-------------------L-------------------R------L-------------------G-------R--------------------------------------------------------D---D-----------
SHIV1157.w214.07 ------------------------------------------------------E-------------------L-------------------R------L-------------------G-------R--------------------------------------------------------D---D-----------
SHIV1157.w214.08 ------------------------------------------------------E-------------------L-------------------R------L-------------------G-------R--------------------------------------------------------D---D-----------
SHIV1157.w214.09 ------------------------------------------------------E-------------------L-------------------R------L-------------------G-------R--------------------------------------------------------D---D-----------
SHIV1157.w214.10 ------------------------------------------------------E-------------------L-------------------R------L---------------------------R-------------------------------------------------------R----D--.--------
SHIV1157.w214.11 ------------------------------------------------------E-------------------L-------------------R------L---------------------------R--------------------------------------------------------D---D--.--------
SHIV1157.w214.12 ------------------------------------------------------E-------------------L-------------------R------L---------------------------R-------------------------------------------------------R----D--.--------
SHIV1157.w214.13 ------------------------------------------------------E-------D-----------L-------------------R------L-------------------G-------R--------------------------------------------------------D---D-----------
SHIV1157.w214.14 ------------------------------------------------------E-------D--N--------L-------------------R------L-------------------G-------R-----------------------------------------------N-HM....D---D-----------
SHIV1157.w214.15 ------------------------------------------------------E-------------------L-------------------R------L--------------------D------RI---------------------------------------------------N---D---D--.--------
SHIV1157.w214.16 ------------------------------------------------------E-------------------L-------------------R------L-------------------G-------R-K------------------------------------------------------D---D--.--------
SHIV1157.w214.17 ------------------------------------------------------E---------A---------L-------------------R------L-------------------G-------R-------------------------------------------------------R----D--.--------
SHIV1157.w214.18 ------------------------------------------------------E---------A----------------------------R------L-------------------GD------R-----------------------V-------------------------------R----D--.--------
SHIV1157.w214.19 ------------------------------------------------------E-------------------L----G--------------R------L--------------------D------R-------------------------------------------------------R----D--.--------
SHIV1157.w214.20 ------------------------------------------------------E-------D-----------L-------------------R------L----------S----T---G--T-P--G---------L--P---N-----V---P-----G----------N----P------D----P--P-------
SHIV1157.w214.21 ------------------------------------------------------E-------------------L-------------------R------L-------------------G-------R--------------------------------------------------------D-G-D-----------
SHIV1157.w214.22 ------------------------------------------------------E-------------------L-------------------R------L--------------------D------R--------------------------------------------------------D---D--.--------
SHIV1157.w214.23 ------------------------------------------------------E-------------------L-------------------R------L------------------R--------R-------------------------------------------------------R----D--.--------

SHIV1157.w350.01 ------------------------------------------------------E-------------------L-KA---------------R------L-------------------KD--N---Q-M------------A-R---------------------------------------IYIDA--D--.--------
SHIV1157.w350.02 ------------------------------------------------------E-------------------L-----------S-------R------L---------------------------R--E-----------A-R------------------------------------G--IYIDA--D--.--------
SHIV1157.w350.03 ------------------------------------------------------E-------------------LKD----------------R------L-------------------KG--N---Q-M------------A-R---------------------------------------IYIDA--D--.--------
SHIV1157.w350.04 ------------------------------------------------------E-------------------L-------------------R------L------------------R----N--RR--E-----------A-R---------------------------------------IYIDA--D--.--------
SHIV1157.w350.05 ------------------------------------------------------E-------------------L-KA---------------R------L-------------------KD--N---Q-M------------A-R------------------------------------G--IYIDA--D--.--------
SHIV1157.w350.06 ------------------------------------------------------E-------------------LKD----------------R------L-------------------KG--N---Q-M------------A-R---------------------------------------IYIDA--D--.--------
SHIV1157.w350.07 ------------------------------------------------------E-------------------L-------------------R------L------------------R----NA--R-M------------A-R-----------------------------E-----G-----D-G-D--.--------
SHIV1157.w350.08 ------------------------------------------------------E-------------------L-KAI--------------R------L-------------------KD--N---Q-M------------A-R---------------------------------------IYIDA--D--.--------
SHIV1157.w350.09 ------------------------------------------------------E-------------------LKD----------------R------L-------------------KG--N---Q-M------------A-R------------------------------------G--IYIDA--D--.--------
SHIV1157.w350.10 ------------------------------------------------------E-------------------L-KAI--------------R------L-------------------KD--N---Q-M------------A-R---------------------------------------IYIDA--D--.--------
SHIV1157.w350.11 ------------------------------------------------------E-------------------L-KA---------------R------L-------------------KD--N---Q-M------------A-R---------------------------------------IYIDA--D--.--------
SHIV1157.w350.12 ------------------------------------------------------E-------------------L-------------------R------L--------------------D------R--E-----------A-R------------------------------------G--IYIDA--D--.--------
SHIV1157.w350.13 ------------------------------------------------------E-------------------L-------------------R------L------------------RG---NA--R-M------------A-R------------------------------------G----IDA--D--.--------
SHIV1157.w350.14 ------------------------------------------------------E-------------------L-------------------R------L--------------------D------R--E-------S---A-R------------------------------------G--IYIDA--D--.--------
SHIV1157.w350.15 ------------------------------------------------------E-------------------L-KA---------------R------L-------------------KD--N---Q-M------------A-R---------------------------------------IYIDA--D--.--------
SHIV1157.w350.16 ------------------------------------------------------E-------------------L-KA---------------R------L-------------------KD--N---Q-M----G-------A-R---------------------------------------IYIDA--D--.--------
```

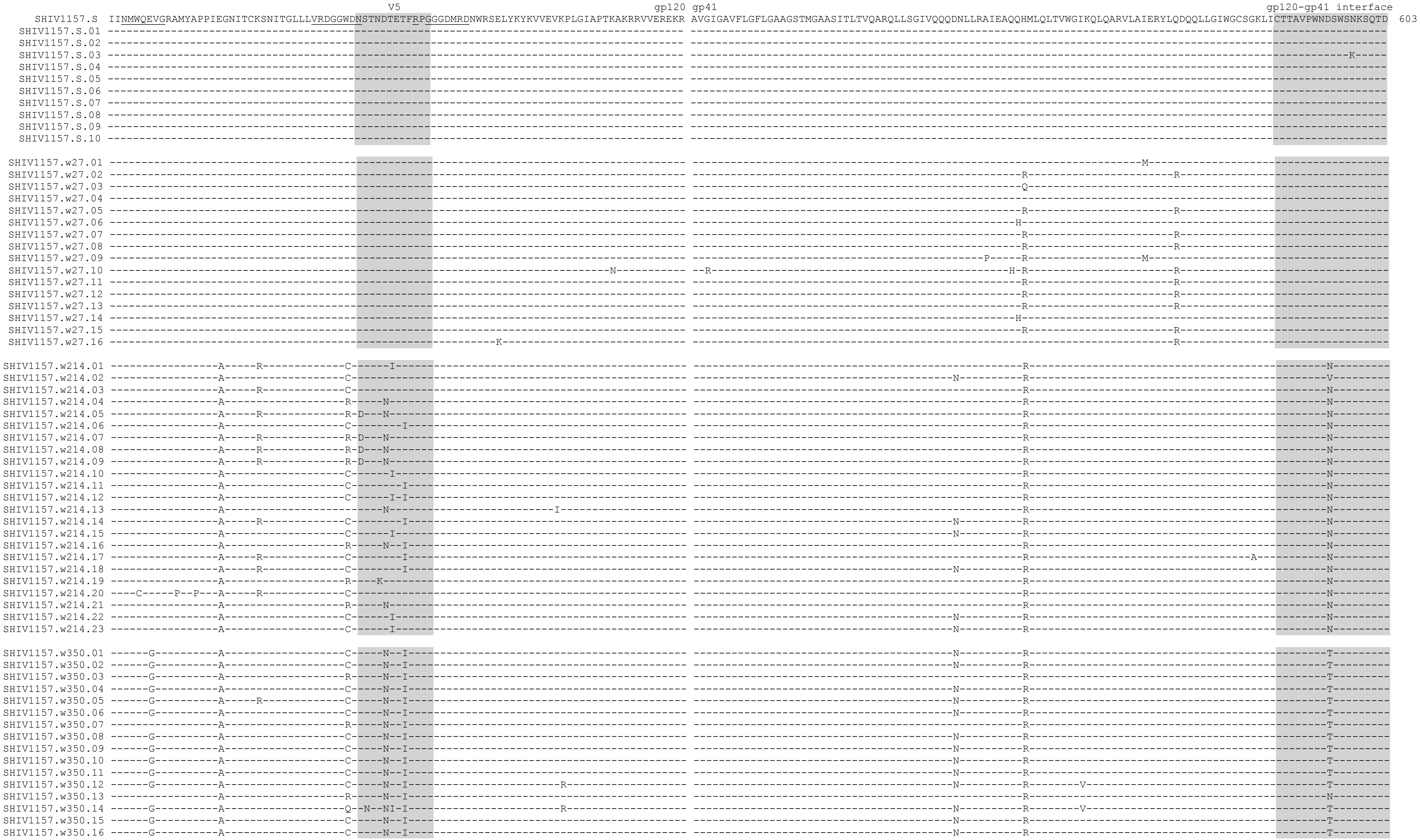

V5
gp120 gp41
gp120-gp41 interface
SHIV1157.S IINMWQEVGRAMYAPPIEGNITCKSNITGLLLVRDGGWDNSTNDTETFRPGGGDMRDNWRSELYKYKVVEVKPLGIAPTKAKRRVVEREKR AVGIGAVFLGFLGAAGSTMGAASITLTVQARQLLSGIVQQQDNLLRAIEAQQHMLQLTVWGIKQLQARVLAIERYLQDQQLLGIWGCSGKLICTTAVPWNDSWSNKSQTD 603
SHIV1157.S.01
SHIV1157.S.02
SHIV1157.S.03
SHIV1157.S.04
SHIV1157.S.05
SHIV1157.S.06
SHIV1157.S.07
SHIV1157.S.08
SHIV1157.S.09
SHIV1157.S.10
SHIV1157.w27.01
SHIV1157.w27.02
SHIV1157.w27.03
SHIV1157.w27.04
SHIV1157.w27.05
SHIV1157.w27.06
SHIV1157.w27.07
SHIV1157.w27.08
SHIV1157.w27.09
SHIV1157.w27.10
SHIV1157.w27.11
SHIV1157.w27.12
SHIV1157.w27.13
SHIV1157.w27.14
SHIV1157.w27.15
SHIV1157.w27.16
SHIV1157.w214.01
SHIV1157.w214.02
SHIV1157.w214.03
SHIV1157.w214.04
SHIV1157.w214.05
SHIV1157.w214.06
SHIV1157.w214.07
SHIV1157.w214.08
SHIV1157.w214.09
SHIV1157.w214.10
SHIV1157.w214.11
SHIV1157.w214.12
SHIV1157.w214.13
SHIV1157.w214.14
SHIV1157.w214.15
SHIV1157.w214.16
SHIV1157.w214.17
SHIV1157.w214.18
SHIV1157.w214.19
SHIV1157.w214.20
SHIV1157.w214.21
SHIV1157.w214.22
SHIV1157.w214.23
SHIV1157.w350.01
SHIV1157.w350.02
SHIV1157.w350.03
SHIV1157.w350.04
SHIV1157.w350.05
SHIV1157.w350.06
SHIV1157.w350.07
SHIV1157.w350.08
SHIV1157.w350.09
SHIV1157.w350.10
SHIV1157.w350.11
SHIV1157.w350.12
SHIV1157.w350.13
SHIV1157.w350.14
SHIV1157.w350.15
SHIV1157.w350.16

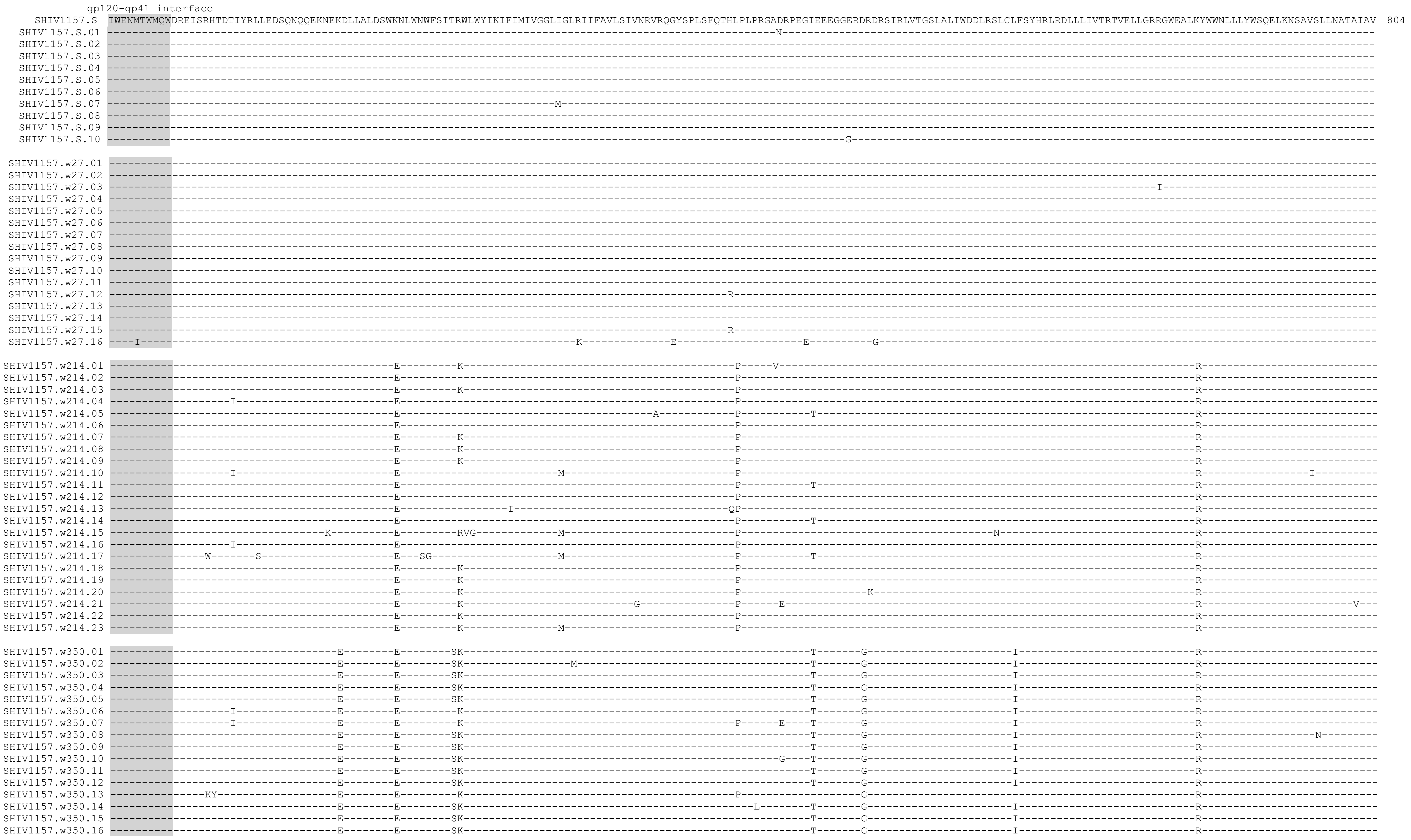
gp120-gp41 interface
SHIV1157.S IWENMTWMQWDREISRHTDTIYRLLEDSQNQQEKNEKDLLALDSWKNLWNWFSITRWLWYIKIFIMIVGGLIGLRIIFAVLSIVNRVRQGYSPLSFQTHLPLPRGADRPEGIEEEGGERDRDRSIRLVTGSLALIWDDLRSLCLFSYHRLRDLLLIVTRTVELLGRRGWEALKYWWNLLLYWSQELKNSAVSLLNATAIAV 804
